# Chinese herbal medicine Shenqi compound for early intervention in patients at high cardiovascular risk of type 2 diabetes mellitus: the protocol of a multicenter, randomized, double-blind, placebo-controlled trial

**DOI:** 10.3389/fcvm.2023.1290240

**Published:** 2024-01-08

**Authors:** Yulin Leng, Zehua Zhang, Nairong Yao, Xiaoxu Fu, Hongyan Xie, Hong Gao, Chunguang Xie

**Affiliations:** ^1^ Hospital of Chengdu University of Traditional Chinese Medicine, Chengdu, Sichuan, China; ^2^ Traditional Chinese Medicine Regulating Metabolic Diseases Key Laboratory of Sichuan Province, Chengdu, Sichuan, China

**Keywords:** type 2 diabetes mellitus, cardiovascular risk, randomized controlled trial, traditional Chinese medicine, Shenqi compound

## Abstract

**Introduction:**

Reducing multiple cardiovascular risk factors is a key link and a challenging clinical problem to reduce the risk of cardiovascular complications and death in patients with diabetes. Currently, there is a lack of clinical studies on patients with diabetes combined with multiple risk factors. Traditional Chinese medicine is believed to have therapeutic effects that contribute to the comprehensive control of multiple cardiovascular factors. This study aims to provide evidence for the efficacy and safety of Shenqi compound (SQC) for early intervention in diabetic patients at high cardiovascular risk.

**Methods and analysis:**

This study is a multicenter, randomized, double-blind, placebo-controlled trial. A total of 120 diabetic patients with high cardiovascular risk were enrolled in five research centers. After a 2-week run-in period, the intervention group received basic treatment and SQC granules, and the control group received basic treatment and placebo granules for a total of 24 weeks, with a 24-week follow-up. The endpoint outcomes are major adverse cardiovascular events and renal-related and peripheral vascular disease events. The primary efficacy outcome is carotid intima-media thickness, and the secondary efficacy outcomes are carotid shear stress, indicators of glucose and lipid metabolism, pancreatic islets function, hemorheology, traditional Chinese medicine syndrome score, and quality of life scale. Safety indicators and adverse events were used to assess the safety of SQC.

**Discussion:**

This study comprehensively evaluated the efficacy and safety of SQC for early intervention in diabetic patients at high cardiovascular risk from the aspects of overall metabolic level, structure, and function of blood vessels, quality of life, and long-term follow-up of endpoint events, providing evidence-based evidence for the short-term efficacy and long-term benefits of early treatment to reduce the risk of diabetic cardiovascular complications.

**Trial Registration:** This trial is registered in the Chinese Clinical Trial Registry on March 9, 2023, https://www.chictr.org.cn/showproj.html?proj=192803 (No. ChiCTR2300069219).

## 1 Introduction

Global diabetes prevention and control strategies have gradually shifted the focus from glycemic control to improving cardiac and renal outcomes (1). Macrovascular disease, mainly atherosclerotic cardiovascular disease (ASCVD), is a major complication and cause of death in patients with type 2 diabetes mellitus (T2DM). The CAPTURE study has shown that the prevalence of cardiovascular disease (CVD) in people with T2DM is 34.8%, with the majority of cases being ASCVD (2). In total, 66.3% of patients with T2DM die from CVD (3). T2DM has been proven to be a CVD risk equivalent (4). The risk of CVD is two to four times higher in diabetic than non-diabetic people (5). The National Cholesterol Education Program shows that patients with T2DM have a more than 20% risk of developing coronary heart disease for the first time within 10 years (6, 7). Therefore, it is a key intervention to delay the deterioration of diabetes complications and reduce mortality to protect the vascular health of diabetic patients as early as possible to maximize cardiovascular benefits.

These risk factors for CVD in patients with T2DM include dyslipidemia, hypertension, obesity, overweight, smoking, and so on. Patients with T2DM often have a combination of these risk factors, which contributes to their high risk of CVD (8). The UKPDS study has shown that the risk factors for coronary artery disease in patients with T2DM are in the order of dyslipidemia, hypertension, hyperglycemia, and smoking (9). Combined control of multiple risk factors can reduce the risk of ASCVD and death in diabetic patients (10–12). However, due to the complexity of chronic disease management, these risk factors are difficult to control effectively in practice. For example, the CCMR-3B study showed that 42% of patients with T2DM had abnormal lipid metabolism, but only 55% received lipid-regulating therapy (8). On the other hand, multiple drug combinations lead to potential side effects, treatment burden, and cost, which can also limit clinical treatment. The trickier question is how to choose the rational clinical treatment options that should maximize benefit. A study has shown that intensive blood glucose control has a limited effect on reducing the risk of CVD and death in patients with T2DM, especially in those with a longer course of disease, older age, and a history of CVD or multiple cardiovascular risk factors (13). The Action to Control Cardiovascular Risk in Diabetes Blood Pressure study has shown that intensive blood pressure control in patients with T2DM did not reduce the total major atherosclerotic cardiovascular events but increased adverse events (14). In conclusion, how to reduce cardiovascular risk factors in this high-risk group is a challenging problem that needs to be addressed in the management of diabetes.

In China, a large number of diabetic patients are at high risk of CVD. Traditional Chinese medicine (TCM) has a wealth of experience in treating diabetes and macrovascular complications, with a variety of treatment methods including herbal compounds, acupuncture, and Tai Chi. We have conducted a meta-analysis of the efficacy and safety of Chinese herbal medicine as an add-on therapy for T2DM patients with carotid atherosclerosis and found that the combination of Chinese herbal medicine may have more advantages than Western medicine alone, which can further reduce carotid intima-media thickness (CIMT) and carotid plaque crouse score, regulate glucose and lipid metabolism, improve insulin resistance, and enhance islet *β*-cell function (15). However, due to a lack of high-quality clinical trials, the low level of evidence for existing treatment options makes it difficult to promote them widely. Shenqi compound (SQC) is a diabetes medicine used in China for more than 20 years and consists of a number of herbs, as shown in Table 1. A series of studies on the production, extraction methods, and active ingredients of SQC granules have been carried out previously (16–18). Clinical trials have shown that SQC has multiple regulatory benefits in treating diabetic patients by lowering blood glucose, regulating lipids, improving insulin resistance, regulating oxidative stress levels, and reducing carotid intima thickness (19, 20). Further animal studies have shown that SQC can reduce glucose variability, ameliorate gut mucosal barrier damage, regulate gut microbiota and metabolites, protect pancreatic islet function in Goto-Kakizaki rats, and ameliorate vascular damage through mechanisms related to improved apoptosis, oxidative stress, and inflammatory microenvironment in the thoracic aorta (21–24). It also showed significant vasoprotective effects in diabetic mice and was superior to rosiglitazone (25). SQC has shown good cardiovascular protective effects and has been included in the *TCM Diagnosis and Treatment Protocol for T2DM* by the China National Clinical Research Base of TCM for diabetes. There is an urgent need for a large sample, multicenter randomized controlled trial to obtain high-quality evidence-based proof for patients with diabetes who are at high risk of CVD.

**Table 1:** Chinese name, Latin name, and species name of each Chinese medicine herb and content in Shenqi compound.

Chinese name	Latin name	Species name	Content in SQC (g)
Renshen	Ginseng Radix et Rhizoma	*Panax ginseng* C.A.Mey.	15
Huangqi	Astragali Radix	*Astragalus mongholicus* Bunge	15
Dihuang	Rehmanniae Radix	*Rehmannia glutinosa* (Gaertn.) DC.	10
Shanyao	Dioscoreae Rhizoma	*Dioscorea oppositifolia L.*	10
Shanzhuyu	Corni Fructus	*Cornus officinalis* Siebold and Zucc.	10
Tianhuafen	Trichosanthis Radix	*Trichosanthes kirilowii* Maxim.	10
Danshen	Salviae Miltiorrhizae Radix et Rhizoma	*Salvia miltiorrhiza* Bunge	10
Dahuang	Rhei Radix et Rhizoma	*Rheum officinale* Baill.	6

Adopted from the Pharmacopoeia of the People's Republic of China 2020 and http://mpns.kew.org/mpns-portal/.

This trial focuses on diabetic patients at high cardiovascular risk and provides evidence-based evidence of the efficacy and safety of SQC in their treatment, provides high-quality evidence-based evidence of the short-term efficacy and long-term benefits of early intervention in TCM in diabetic patients at high cardiovascular risk, and provides solutions to the cardiovascular benefit needs of clinical diabetes management and control.

## 2 Methods and analyses

### 2.1 Study design

This study is a multicenter, randomized, double-blind, placebo-parallel controlled clinical trial. Changes in outcomes from baseline, the CIMT, glycometabolism, lipid metabolism, islet function, hemorheology, carotid ultrasound, carotid shear force, TCM syndrome score, and life quality score to provide evidence-based evidence of the efficacy and safety of early intervention of SQC in diabetic patients at high cardiovascular risk.

In this trial, 120 patients with T2DM at high cardiovascular risk were enrolled in five centers. After a 2-week run-in period, participants will be randomized 1:1 to receive SQC or placebo for 24 weeks. The trial flow is shown in Supplementary Figure S1, and the schedule is summarized in Supplementary Table S1 (see Additional File 1). Randomization, allocation concealment, blinding, and data analysis will be entrusted to external experts from the Chinese Evidence-based Medicine Center (the Chinese Cochrane Center), who are not involved in the clinical conduct of this trial. They will act as data monitors and conduct local and email monitoring every 3 months. This study design is in accordance with the Consolidated Standards of Reporting Trials (CONSORT) extension for Chinese herbal medicine formulas and Standard Protocol Items for Clinical Trials with TCM (SPIRIT-TCM Extension 2018) (26, 27).

### 2.2 Inclusion criteria

1. According to the *Guideline for the Prevention and Treatment of T2DM in China* published by the Chinese Diabetes Society, patients diagnosed with T2DM were enrolled (5).
2. According to the *Guiding Principles for New Drug Clinical Research of TCM*, the *Guidelines for Prevention and Treatment of Diabetes in TCM*, and the diagnostic scale for Qi and Yin deficiency syndrome of diabetes, patients who meet the diagnostic criteria for TCM Qi and Yin deficiency syndromes have the following symptoms: thirst, fatigue and weakness, bulimia, talking laziness, heat in palms, chest, and soles, red tongue with thin white dry fur, and thin and weak pulse (28–30).
3. Patients who were between the ages of 18 and 65 years were included.
4. Patients with CIMT ≥ 1 mm on color Doppler ultrasound were included.
5. Patients who had at least two cardiovascular risk factors (31):
  (1) Hypertension: patients who had been diagnosed with hypertension and current blood pressure ≥ 130/80 mmHg when in an unmedicated state.
  (2) Dyslipidemia: patients who had low-density lipoprotein cholesterol (LDL-C) > 2.6 mmol/L and at least one of the following conditions: non-HDL-C > 3.4 mmol/L, total cholesterol (TC) > 4.5 mmol/L, triglycerides (TG) > 1.7 mmol/L, or high-density lipoprotein cholesterol (HDL-C) < 1.04 mmol/L.
  (3) Obesity/overweight: patients who had body mass index (BMI) ≥ 24 kg/m^2^ or central obesity (waist circumference ≥90 cm for men and ≥85 cm for women).
  (4) Smoking history: patients who have smoked continuously or cumulatively for more than 6 months and are still currently smoking.
  (5) Patients who were ≥ 40 years old.
  (6) Patients for whom the course of diabetes has been more than 5 years.
6. Patients who participated voluntarily and signed the informed consent form.

### 2.3 Exclusion criteria

1. Patients who have had an acute metabolic disorder, such as diabetic ketoacidosis, or stringent state in the month before the screening.
2. Patients whose previous coronary angiography confirmed the diagnosis of coronary heart disease, whose electrocardiogram or clinical symptoms suggested coronary heart disease, or whose previous cerebrovascular accident was confirmed, or whose vascular ultrasound showed peripheral arterial plaque formation or arterial stenosis or occlusion.
3. Patients with impaired liver function (serum alanine aminotransferase or aspartate aminotransferase or alkaline phosphatase greater than 1.5 times the upper limit of normal value), impaired renal function [serum creatinine greater than the upper limit of normal value or estimated glomerular filtration rate (eGFR) ≤ 60 mL·min^−1^·(1.73 m^2^)^−1^ at screening].
4. Patients with malignant tumors, severe mental illness, or serious diseases whose life expectancy is less than 2 years.
5. Women who were planning to become pregnant or who were pregnant or breastfeeding.
6. Patients with a known allergy to the herbs in the SQC.
7. Patients who have participated in other clinical trials within the last 3 months.
8. The researcher judges that the patient is unsuitable for this trial due to poor compliance.

### 2.4 Dropout criteria

1. Participants who voluntarily withdrew.
2. The participant's serum alanine aminotransferase or aspartate aminotransferase is three times greater than the upper limit of normal value, and it is still abnormal after repeat testing within 1 week.
3. The participant's serum creatinine is 1.5 times greater than the upper limit of normal value, and it is still abnormal after repeat testing within 1 week.
4. Participants who do not take the study medication as prescribed; that is, the dose rate is less than 80% or greater than 120%.
5. Participants violate this protocol during the trial by using drugs that are outside the prescribed range and interfere with the efficacy evaluation.
6. Participants who are lost to follow-up without explanation.
7. Participants who are unblinded.

### 2.5 Recruitment

Participants were recruited from the Hospital of Chengdu University of TCM (Chengdu, China), the Tongji Hospital of Tongji Medical College of Huazhong University of Science and Technology (Wuhan, China), the Affiliated TCM Hospital of Southwest Medical University (Luzhou, China), the Chongqing TCM Hospital (Chongqing, China), and the Affiliated Hospital of Shanxi University of TCM (Xianyang, China). All these institutions are Good Clinical Practice Centers approved and accredited by the China National Medical Products Administration.

### 2.6 Randomization

This trial was randomized using central stratified block randomization. The PROC PLAN procedure statement of SAS statistical software was used to prespecify the center number, set the seed number, stratified by center, set the number of blocks and block length, and simulate the generation of random sequence numbers for the interventions received by participants. The study drug was packaged in a random sequence. Participants were enrolled sequentially into groups according to the order of random sequence numbers.

### 2.7 Allocation concealment

Staff blinded the drugs according to the number of centers, grouping sequences, and sequential sequences generated by the random blocks. After blinding, the drugs could not be distinguished by their sequential number. The blind code is a six-column sequential allocation list, where the first column is the center number, the second column is the block group number, the third column is the block length, the fourth column is the participant's serial number, the fifth column is the randomized group identification, and the sixth column is the corresponding allocated number. This list is kept in duplicate, sealed, and locked in a lightproof envelope by the staff who generated the random sequence and by the staff of the pharmaceutical company.

### 2.8 Blinding

In this study, participants, researchers, outcome assessors, data collection and monitoring staff, and data management and statistical analysis staff were blinded. Drug administrators were not involved in the trial. The whole process was supervised by experts from the Chinese Evidence-based Medicine Center.

SQC and placebo were both produced by the Sichuan Neo-Green Pharmaceutical Technology Development Co. Ltd. (Chengdu, China). The ingredients of the placebo were maltodextrin, bitters, and food colorants, which comply with food safety regulations. The smell, taste, appearance, packaging, dosage form, production batch number, and date of the placebo were identical to those of SQC granules. Based on the previous study experience of the research group, this placebo formulation has the feasibility of a blinding method.

Primary unblinding of the statistical analyst was performed after the data lock, and secondary unblinding was performed after the statistical analysis. Each center received the emergency unblinding letter with study drug information according to the drug allocation. Unblinding may occur in an emergency (serious adverse event, suspected unexpected serious adverse reaction). The participants were treated according to the drug information, and the time, date, and reason for unblinding were recorded. All emergency envelopes must be kept with the trial records at the end of the trial in the clinical research unit.

### 2.9 Interventions

#### 2.9.1 Run-in period

To standardize basic treatment and ensure compliance, participants entered a 2-week run-in period after screening. They received individualized basic treatment for glucose lowering, blood pressure control, lipid management, and antiplatelet therapy in accordance with the guidelines (5, 31). They received the same lifestyle and behavior education on diabetes self-management, diet, and daily physical activity.

#### 2.9.2 Intervention period

Participants were randomized to intervention and control groups: (1) intervention group: basic treatment and SQC granules; and (2) control group: basic treatment and placebo granules. Both SQC and placebo specifications were 15 g/piece. Dosage indication was three times daily, before meals. For drug administration, granules were dissolved in 100 mL of hot water, stirred, covered, sealed, and taken orally between 30°C and 37°C. The intervention period was 24 weeks.

During this time, participants were not allowed to use acupuncture, massage, or other TCM interventions outside of this study and were also not allowed to use medications with cardiovascular risks.

### 2.10 Outcomes

Primary and secondary efficacy outcomes were measured once pretreatment and once post-treatment for a total of two times. Endpoint outcomes were measured once pre-treatment, once post-treatment, and once at 24 weeks of follow-up, for a total of three times.

#### 2.10.1 Primary efficacy outcomes

##### 2.10.1.1 CIMT

Equipment: The color Doppler ultrasound machine used uniformly in all study centers was the Philips Diagnostic Ultrasound System and Transducers EPIQ 7 (Philips, Inc.). The specific sites for CIMT measurements were the distal common carotid artery (1.0–1.5 cm below the level of the bifurcation) and the carotid bulb (the relative extension of the beginning of the internal carotid artery).

#### 2.10.2 Secondary efficacy outcomes

1. Glycometabolism outcome: fasting plasma glucose (FPG) and hemoglobin A1c levels.
2. Lipid metabolism outcome: TC, TG, LDL-C, and HDL-C levels.
3. Islet function outcome: fasting insulin (FINS), calculated by HOMA-*β* = 20 × FINS × (FPG-3.5), HOMA-IR = FPG × FINS/22.5.
4. Hemorheology outcome: plasma viscosity, erythrocyte aggregation index, hematocrit value, and erythrocyte sedimentation rate.
5. Carotid ultrasound: plaque and degree of stenosis, end-diastolic arterial internal diameter (mm), peak systolic velocity (PSV, cm/s), end-diastolic velocity (EDV, cm/s), and mean velocity (Vm, cm/s) in the carotid artery. The following parameters were calculated: pulse index PI = (PSV−EDV)/Vm, resistance index RI = (PSV−EDV)/PSV, and S/D = PSV/EDV.
6. Carotid artery shear stress: *τ* = 4 × *μ* × PSV/D (where *μ* is the plasma viscosity and *D* is the end diastolic arterial internal diameter).
7. TCM syndrome score (28).
8. Life quality score: (1) Quality of life scale for patients with T2DM (DMQLS) and (2) the Chinese version of the Diabetes Management Self-Efficacy Scale (C-DMSES).

#### 2.10.3 Endpoint outcomes

This trial evaluates two endpoint outcomes: the incidence of endpoint events and the time to progress to endpoint events.

Endpoint events are defined as follows: (1) progression of peripheral vascular disease, defined as carotid plaque (limited IMT ≥ 1.5 mm), stenosis (degree ≥ 50%), or occlusion; (2) major adverse cardiovascular events: cardiovascular death, myocardial infarction, ischemic stroke, and hospitalization due to heart failure; and (3) renal-related events: urinary microalbumin creatinine ratio and eGFR.

### 2.11 Follow-up

After the end of the treatment period, the study was followed for a further 24 weeks to observe endpoint events.

### 2.12 Safety evaluation

Safety outcomes included the following: (1) body temperature, respiration, heart rate, and blood pressure, which were monitored at each 4th week of outpatient visit; and (2) blood, urine, and stool routine tests, serum liver function (alanine aminotransferase, aspartate aminotransferase, total bilirubin, alkaline phosphatase, gamma-glutamyl transferase), and renal function (blood urea nitrogen, creatinine) tests, and 12-lead electrocardiography examination, which were performed once before the intervention, once at 4 weeks, once at 12 weeks, and once at 24 weeks after the intervention, for a total of four times. Adverse events (AEs) were monitored throughout the treatment period and graded according to the Common Terminology Criteria for Adverse Events version 5.0.

The trial may be terminated early in the presence of clustered serious safety concerns during the trial, supported by evaluative evidence related to the intervention.

### 2.13 Data collection and management

Baseline data included the following: (1) demographic data: gender, age, height, weight, waist circumference, hip circumference, calculated BMI, and waist–hip ratio; (2) medical history of diabetes and other diseases; and (3) menstrual history of women of childbearing age; suspected pregnancy should be checked by a urine pregnancy test.

Blood and urine samples were collected on an empty stomach at the hospital in the morning. Test methods and equipment are standardized in each center. The drug package recovery counting method, combined with the medication self-assessment form completed by the participants, was used to monitor the drug-taking rate. A dosing rate of 80%–120% was considered effective dosing.

In this study, paper Case Record Form was used to collect data, and EpiData software V3.1 was used for data management. Two data entry staff entered the data independently using the data verification function for double-checking. After entry, the manual verification plan based on the data verification plan was used to screen for questions. Database locking was performed by a study leader, a statistical analyst, and a data administrator upon signature of the database lock record. REC files were generated, and CHK files were created and exported in Excel for statistical analysis.

### 2.14 Sample size

This study is a superiority trial using CIMT as the primary outcome for sample size estimation compared to a placebo. Based on published clinical studies, the CIMT mean for the control group on long-term placebo treatment was assumed to be 1.12 mm (32). In this trial, the CIMT mean of the intervention group was estimated to be 1.03 mm, the standard deviation of the two groups was set to 0.10, the superiority value was set to 0.03, and the one-sided test was set to *α* = 0.025, 1-*β* = 0.8. The two groups were randomized in a 1:1 ratio. The minimum sample size for each group was calculated using PASS 15.0 software to obtain 45 cases, for a total of 90 cases. Considering the dropout rate and the balance between the two groups and five centers, a total sample size of 120 participants was required, with 60 participants in each group and 24 participants in each center.

### 2.15 Data analysis

Statistical analysis included baseline data, endpoint, efficacy, and safety outcomes using SPSS 26.0 and SAS 9.1 software. *P*-value < 0.05 was considered statistically significant. Data files were locked after blinded review and verified as reliable and error-free, and the analysis process was secondary unblinding.

The full analysis set (FAS) was selected for analysis of demographic and other baseline characteristics for comparability analysis. For efficacy and endpoint results, intention-to-treat analysis was mainly used primarily for FAS and also for the per-protocol set (PPS). Differences in results between the two datasets were compared, and if there were inconsistencies, the cause was identified. The safety set (SS) analysis was performed on participants who had received the study drugs at least once after randomization and who had experienced AE.

The Cochran–Mantel–Haenszel test was used for qualitative data, and analysis of variance and analysis of covariance were used for quantitative data. The log-rank test was used to assess the difference in incidence and to plot a curve for the endpoint outcome. The Cox proportional hazards regression model was used for multifactorial analysis. Subgroup analysis was conducted to explore differences in intervention efficacy among different populations based on age (>50 or ≤50 years), gender, smoking history, hypertension, hyperlipidemia, and drug combination (statin use or not, ACEI/ARB drugs ≥50% of the target dose).

If participants withdrew from the study, they were asked to return for a final visit, if possible, and their data were used using the last observation carried forward (LOCF) method. Participants who took the study drug and had a baseline follow-up visit were included in the FAS for analysis, and those who had an AE were included in the SS for analysis. If the rate of missing data was high (≥20%) and the variable containing the missing value was important for the question under study, the missing data were analyzed using the multiple imputation method.

## 3 Discussion

The carotid artery is a common site of atherosclerosis, with lesions often appearing earlier than in the coronary and cerebral arteries. Clinical studies have shown that a reduction in CIMT reduces the risk of cardiovascular events; therefore, CIMT progression is considered an effective surrogate marker for cardiovascular events and can predict cardiovascular risk in relatively low-risk populations (33, 34). On the other hand, CIMT screening allows cardiovascular risk stratification in patients with diabetes (35). As a non-invasive test, ultrasound has qualitative and quantitative value in the repeated detection of IMT and plaque changes. Currently, many clinical guidelines recommend using CIMT and plaque characteristics to help predict the risk of macrovascular complications in patients with T2DM (36). Therefore, this study focused on CIMT changes in diabetic patients to assess ASCVD risk reduction.

This study focused on changes in hemorheology and hemodynamics, which are indicators of early changes in vascular structure and function. Hematocrit value and plasma viscosity have been shown to correlate with heart rate variability in patients with T2DM (37). The erythrocyte sedimentation rate has been shown to be a predictor of coronary heart disease and heart failure (38, 39). Increased erythrocyte aggregation leads to an inability to pass through the capillaries, which is an important factor in the development of macroangiopathy in diabetic patients (40). On the other hand, the vessel wall is exposed to prolonged blood flow and the direction of fluid flow in normal vessels is laminar, which has certain anti-inflammatory, antioxidant stress, and antiatherosclerotic effects, while hemodynamic changes have an important effect on the elasticity and contractile function of the vessels, with wall shear stress in the parallel direction being closely relevant to atherosclerosis (41). Vascular endothelial cells, smooth muscle cells, and fibroblasts can sense shear stimulation and convert them into intracellular signals (42–44). Shear stress changes are present in patients with T2DM and atherosclerosis, and shear stress changes on the carotid artery wall in patients with risk factors for atherosclerosis are significantly associated with the number of risk factors such as hyperglycemia, hypertension, and dyslipidemia (45, 46). Metformin has been shown to improve aortic wall shear stress abnormalities in youngsters with type 1 diabetes (47). Pioglitazone has been shown to improve diastolic shear stress of the brachial artery in patients with impaired glucose tolerance (48). However, there is a temporary lack of clinical evidence on the effect of TCM on arterial shear force in diabetic patients. Therefore, carotid artery shear stress is selected in this study to identify early changes in the vascular microenvironment in patients with diabetes.

In efficacy trials for early intervention in chronic diseases, it is difficult to observe sufficient endpoint events during the trial, but there is undeniable value in focusing on the early stages of the disease. The highlight of this study is that patients with T2DM at high cardiovascular risk were selected as the research population, and in response to this contradiction, multiple outcomes such as islet function, vascular structure, and vascular function were selected to evaluate the early efficacy of SQC from multiple perspectives. The shortcoming of this study is that only 24 weeks of intervention and 24 weeks of follow-up were carried out, which is relatively short for the duration of a chronic disease.
