# Supplementary material for: Chinese herbal medicine Shenqi compound for early intervention in patients at high cardiovascular risk of type 2 diabetes mellitus: the protocol of a multicenter, randomized, double-blind, placebo-controlled trial
Source: Front Cardiovasc Med. 2024 Jan 8;10:1290240. doi: 10.3389/fcvm.2023.1290240 (PMC10800938; doi:10.3389/fcvm.2023.1290240)
Supplement: Supplementary file 1 [file Table1.docx]

**Supplementary Table 1** The schedule of study

|  | **Study period** | | | | | | | | | | | | | | | |
| --- | --- | --- | --- | --- | --- | --- | --- | --- | --- | --- | --- | --- | --- | --- | --- | --- |
|  | **Enrolment** | **Run-in** | **Intervention** | | | | | | | | | | | | | **Follow-up** |
| **Time point (weeks )** | **Before** | **-2** | **0** | **2** | **4** | **6** | **8** | **10** | **12** | **14** | **16** | **18** | **20** | **22** | **24** | **48** |
| Outpatient visit | X | X | X |  | X |  | X |  | X |  | X |  | X |  | X |  |
| Telephone vist |  |  |  | X |  | X |  | X |  | X |  | X |  | X |  | X |
| **Enrolment** |  |  |  |  |  |  |  |  |  |  |  |  |  |  |  |  |
| Eligibility | X |  |  |  |  |  |  |  |  |  |  |  |  |  |  |  |
| Informed consent | X |  |  |  |  |  |  |  |  |  |  |  |  |  |  |  |
| Demographics and medical history | X |  |  |  |  |  |  |  |  |  |  |  |  |  |  |  |
| Randomization |  | X |  |  |  |  |  |  |  |  |  |  |  |  |  |  |
| **Intervention** |  |  | X | X | X | X | X | X | X | X | X | X | X | X | X |  |
| **Assessments** |  |  |  |  |  |  |  |  |  |  |  |  |  |  |  |  |
| CIMT, other carotid ultrasound and carotid artery shear stress |  |  | X |  |  |  |  |  |  |  |  |  |  |  | X |  |
| Blood glucose, lipid, islet function, hemorheology tests |  |  | X |  |  |  |  |  |  |  |  |  |  |  | X |  |
| TCM syndrome score |  |  | X |  |  |  |  |  |  |  |  |  |  |  | X |  |
| Life quality score |  |  | X |  |  |  |  |  |  |  |  |  |  |  | X |  |
| Vital signs |  |  | X |  | X |  | X |  | X |  | X |  | X |  | X |  |
| Blood, urine and stool routine tests, liver and renal function tests, ECG |  |  | X |  | X |  |  |  | X |  |  |  |  |  | X |  |
| Endpoint events |  |  | X | X | X | X | X | X | X | X | X | X | X | X | X | X |
| Adverse events |  |  | X | X | X | X | X | X | X | X | X | X | X | X | X |  |

**Notes:** A deviation of ± 3 days is allowed at each time point. **Abbreviations:** CIMT, Carotid intima-media thickness; TCM, traditional Chinese medicine; ECG, electrocardiogram.
