# Supplementary figures and images for: Chinese herbal medicine Shenqi compound for early intervention in patients at high cardiovascular risk of type 2 diabetes mellitus: the protocol of a multicenter, randomized, double-blind, placebo-controlled trial
Source: Front Cardiovasc Med. 2024 Jan 8;10:1290240. doi: 10.3389/fcvm.2023.1290240 (PMC10800938; doi:10.3389/fcvm.2023.1290240)

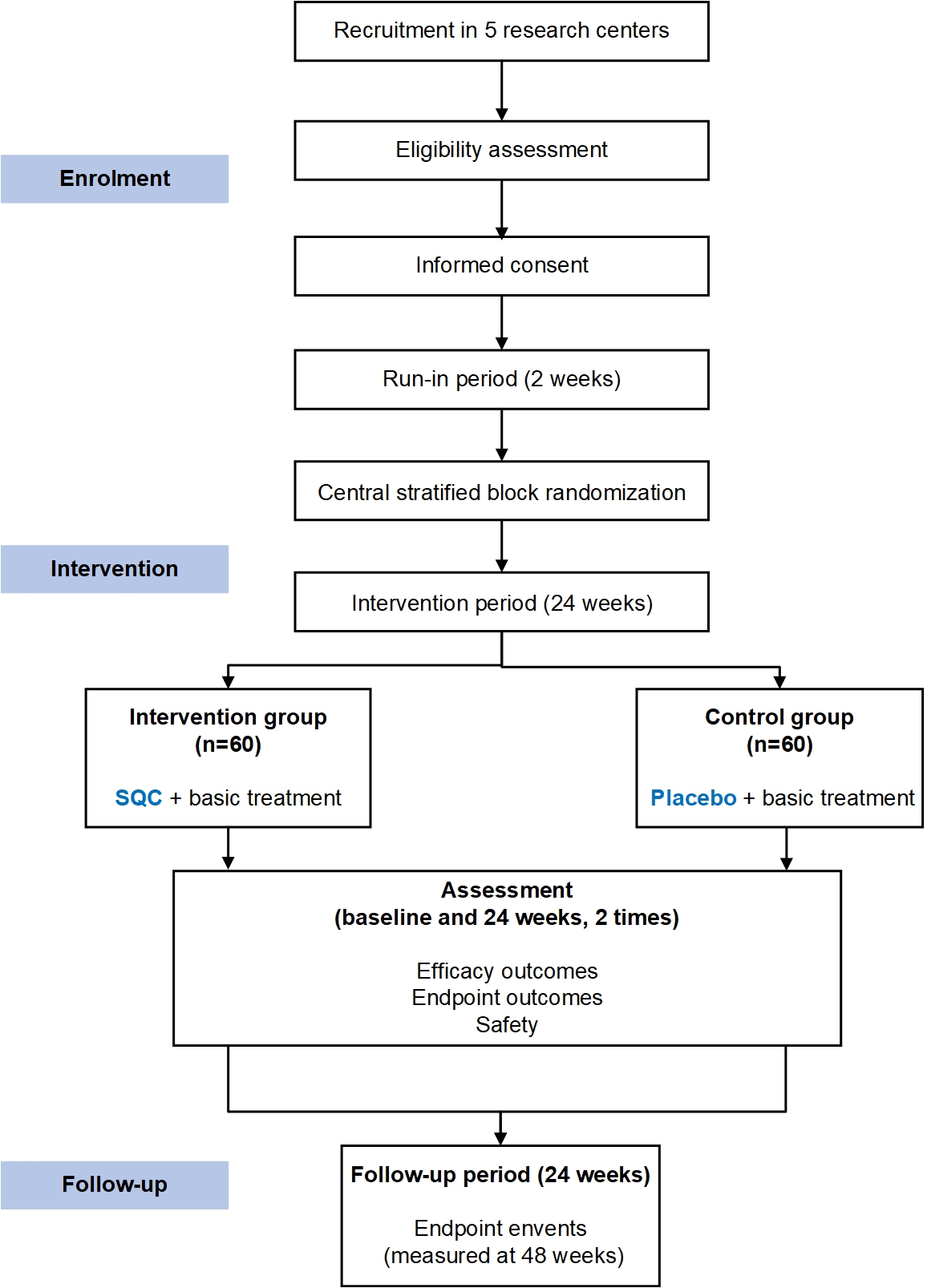

Supplement: Supplementary file 2 [file Image1.jpeg]
